# A Novel Quantum Dots-Based Fluorescent Sensor for Determination of the Anticancer Dacomitinib: Application to Dosage Forms

**DOI:** 10.3390/molecules28052351

**Published:** 2023-03-03

**Authors:** Manal A. Alossaimi, Heba Elmansi, Mai Alajaji, Ali Altharawi, Abdulmalik S. A. Altamimi, Galal Magdy

**Affiliations:** 1 Pharmaceutical Chemistry Department, College of Pharmacy, Prince Sattam Bin Abdulaziz University, Al-Kharj 11942, Saudi Arabia; 2 Pharmaceutical Analytical Chemistry Department, Faculty of Pharmacy, Mansoura University, Mansoura P.O. Box 35516, Egypt; 3 King Abdullah International Medical Research Center, College of Pharmacy, King Saud bin Abdulaziz University for Health Sciences, Riyadh 14611, Saudi Arabia; 4 Pharmaceutical Analytical Chemistry Department, Faculty of Pharmacy, Kafrelsheikh University, Kafrelsheikh P.O. Box 33511, Egypt

**Keywords:** nitrogen-doped carbon quantum dots, dacomitinib, fluorescence, sensor, quenching

## Abstract

One of the most promising drugs recently approved for the treatment of various types of cancer is dacomitinib, which belongs to the tyrosine kinase inhibitor class. The US Food and Drugs Administration (FDA) has recently approved dacomitinib as a first-line treatment for patients suffering from non-small cell lung cancer (NSCLC) with epidermal growth factor receptor (EGFR) mutations. The current study proposes the design of a novel spectrofluorimetric method for determining dacomitinib based on newly synthesized nitrogen-doped carbon quantum dots (N-CQDs) as fluorescent probes. The proposed method is simple and does not require pretreatment or preliminary procedures. Since the studied drug does not have any fluorescent properties, the importance of the current study is magnified. When excited at 325 nm, N-CQDs exhibited native fluorescence at 417 nm, which was quantitatively and selectively quenched by the increasing concentrations of dacomitinib. The developed method involved the simple and green microwave-assisted synthesis of N-CQDs, using orange juice as a carbon source and urea as a nitrogen source. The characterization of the prepared quantum dots was performed using different spectroscopic and microscopic techniques. The synthesized dots had consistently spherical shapes and a narrow size distribution and demonstrated optimal characteristics, including a high stability and a high fluorescence quantum yield (25.3%). When assessing the effectiveness of the proposed method, several optimization factors were considered. The experiments demonstrated highly linear quenching behavior across the concentration range of 1.0−20.0 μg/mL with a correlation coefficient (r) of 0.999. The recovery percentages were found to be in the range of 98.50–100.83% and the corresponding relative standard deviation (%RSD) was 0.984. The proposed method was shown to be highly sensitive with a limit of detection (LOD) as low as 0.11 μg/mL. The type of mechanism by which quenching took place was also investigated by different means and was found to be static with a complementary inner filter effect. For quality purposes, the assessment of the validation criteria adhered to the ICHQ2(R1) recommendations. Finally, the proposed method was applied to a pharmaceutical dosage form of the drug (Vizimpro^®^ Tablets) and the obtained results were satisfactory. Considering the eco-friendly aspect of the suggested methodology, using natural materials to synthesize N-CQDs and water as a diluting solvent added to its greenness profile.

## 1. Introduction

**Figure 1:**
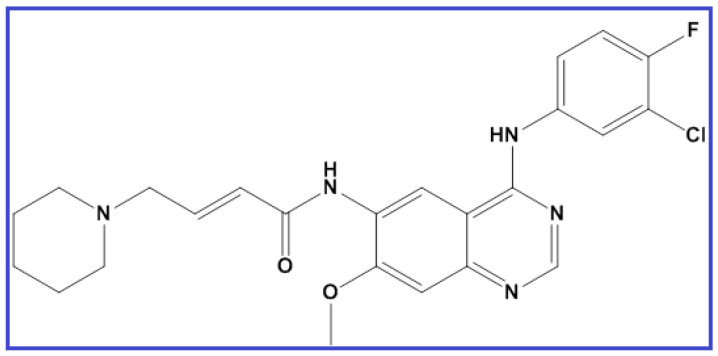
Chemical structure of dacomitinib (DCB).

Cancer in all its types is one of the major causes of death across the world. The most fatal type of cancer is lung cancer, which includes the following two main types: small-cell lung carcinoma (SC) and non-small cell lung carcinoma (NSCLC); the latter one constitutes about 85% of lung cancer cases [1]. Many treatments have been proposed for the management of NSCLC over the last few decades. Among the most recent drugs approved by the United States FDA is dacomitinib (DCB). It is an anilinoquinazoline derivative that belongs to the small-molecule kinase inhibitor (SMKI) class of HER1/EGFR, HER2, and HER4 tyrosine kinases. It is suitable for cases with human epidermal growth factor receptor 2 (HER2) autophosphorylation and tumor growth and is generally administered orally once per day [2]. Commercially, the only approved tablet dosage form of DCB is available under the trade name Vizimpro^®^ and the recommended daily dosage is 45 mg [2]. As depicted in Figure 1, DCB has the molecular formula C_24_H_25_ClFN_5_O_2_, leading to the associated chemical name (2E)-N-[4-[(3-Chloro-4-fluorophenyl)amino]-7-methoxyquinazolin-6-yl]-4-(piperidin-1-yl)but-2-enamide monohydrate [1]. According to the manufacturer, in vitro experiments that applied the Vizimpro^®^ treatment on human tumors implanted in mice showed that the drug is capable of inhibiting the autophosphorylation of EGFR as well as the human epidermal growth factor receptor 2 (HER2) and limiting the growth of the tumor. The mean bioavailability of DCB after oral administration was found to be about 80% [2].

Determining drug contents is an essential part of the quality assurance and development of drug products. To the best of the authors’ knowledge, no spectroscopic methods have been reported for the determination of DCB, which is likely due to its novelty [3]. A survey of the related literature indicated that few analytical methods, including liquid chromatography with mass spectrometry (LC-MS/MS), have been reported for its analysis [1,4]. Two studies proposed methods for the analysis of DCB through LC-MS/MS, using solvents such as DMSO and formic acid. These methods were shown to have satisfactory sensitivity levels. However, when the cost and environmental impact are taken into consideration, DMSO and formic acid may not be preferred. Therefore, it is essential to develop a novel DCB determination method that is simple, economical, and ecofriendly, which motivated the authors to pursue the subject of this study.

Fluorescence spectroscopy is one of the most selective and sensitive techniques with a wide linear range of responses, without affecting precision. In order to take the advantages of the fluorescence technique to the measurement of non-fluorescent drugs, one can use an interface fluorescent molecule, so that the analyte can quantitatively quench the intensity of its fluorescence emission.

The quantum dot (QD) is a small nanoparticle that is a few nanometers in size made from a semiconductor material. QDs have many attractive optical and electronic properties that make them of interest in the nanotechnology and pharmaceutical disciplines, among others. QDs have been used in a variety of recent applications, such as pharmaceutical analysis, drug delivery, diagnosis, and cell labeling [5,6,7]. Comprehensive details of these fields can be found in recent reviews [8,9]. Interestingly, they were reported to have several applications as sensors and probes in spectroscopic methods for the estimation of different pharmaceutical and environmental samples. Spectrofluorimetry is one of the tools used for assessment, which has been widely used with QDs [10]. These fluorescent sensors have the benefit of demonstrating high fluorescence intensity and good selectivity. The different routes for QD synthesis including top-down and bottom-up methods were discussed in detail in the literature [9,10]. Recently, a new type of QD made from carbon instead of semiconductor materials has emerged. These nanomaterials are referred to as carbon QDs (CQDs) and have a number of advantages over standard semiconductor QDs, including a much lower toxicity, a better environmental impact, a lower cost, and more facile synthesis [11].

In accordance with the objectives of this study, non-metallic heteroatom-doped CQDs (HDCQDs) were used, which have been reported to possess superior optical and electrical characteristics, as well as distinctive fluorescence characteristics. The most common doping elements used in CQDs include boron, fluorine, nitrogen, phosphorus, and sulfur [11]. Doping of CQDs with nitrogen can modify their electronic and chemical features. Nitrogen is adjacent to carbon in the periodic table, and thus possesses a similar atomic radius (0.70 Å) to carbon (0.77 Å) and greater electronegativity (χN = 3.04) than carbon (χc = 2.55), making it easier to incorporate nitrogen into carbon networks via substitution doping. Due to the comparable atomic size and five valence electrons available for bonding with carbon atoms, which significantly enhance fluorescence properties, N-doping is the most popular method for enhancing the fluorescent properties of CQDs. The nitrogen atom injects electrons into CQDs, altering the internal electronic environment and producing CQDs with high fluorescence, exceptional catalytic activity, good cell permeability, and minimal cytotoxicity. Subsequently, the superior luminescence characteristic of nitrogen-doped carbon quantum dots (N-CQDs) allows them to be used for biomedical imaging and other optoelectronic applications [9,12].

The fluorescence emissions of CQDs take place through multiple mechanisms, including bandgap transitions and surface defects [13]. One specific type of HDCQDs that has attracted the attention of many researchers is N-CQDs, due to their appealing electrical properties and chemical reactivity, leading to the application of CQDs in different fields [14,15]. 

The current study presents the first spectrofluorimetric-based determination method for DCB without the need for any pre-derivatization steps or high-cost instrumentation. The proposed method is based on the quantitative and selective quenching of the native fluorescence of the prepared N-CQDS upon increasing the concentration of DCB. In the proposed method, N-CQDs were prepared using a rapid, facile, and green microwave-assisted approach in less than 10 min, utilizing orange juice (a carbon source) and urea (a nitrogen source) as economic, ecofriendly, and readily available starting materials. This, in turn, adds an important advantage to the proposed method in terms of commercial viability and sustainability. The proposed synthetic approach in the current study possesses different advantages over the reported methods for the synthesis of CQDs, which may require the utilization of expensive instrumentation, long time, or complicated chemical interactions under drastic conditions, including boiling with concentrated sulfuric acid as is the case in carbonizing organics [16,17,18]. In fact, these complicated preparatory conditions are expensive, time-consuming and will accordingly decrease the method’s greenness. 

Due to the superior features of N-CQDs, the proposed method has a multitude of advantages over the reported LC-MS/MS, including a shorter analysis time, a lower cost, simpler procedures, better availability, biocompatibility, and method greenness. It is important to note that LC-MS is an expensive option, both in terms of capital and operational costs. The instrument requires skilled personnel to set it up.

As will be shown later, applying the proposed method to Vizimpro^®^ tablets produced satisfactory accuracy and precision. This makes the developed method a strong contender for application in the routine analysis of DCB in quality control laboratories.

## 2. Results and Discussion

**Scheme 1:**
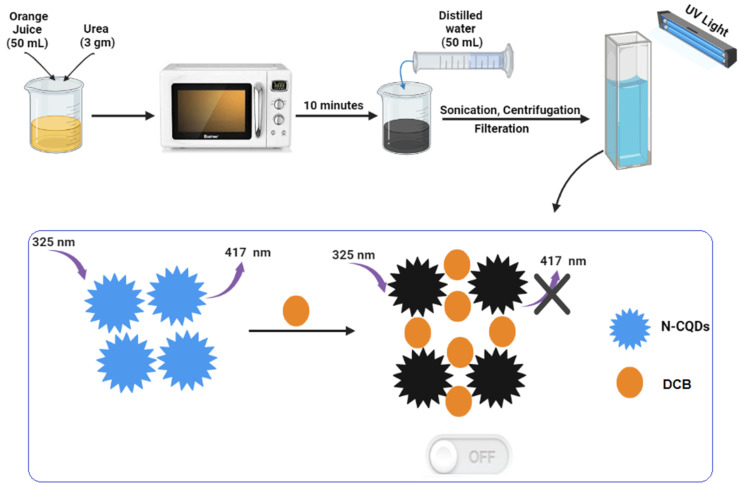
Synthesis of N-CQDs and application for the determination of DCB (Created with BioRender.com, accessed on 28 February 2023).

As mentioned, quantum dots (QDs) can be used as fluorescent probes and offer many benefits and merits over their classical counterparts. They were reported to have superior photoluminescence properties, involve simple and cost-effective synthesis procedures, economical starting materials and high water solubility, biocompatibility, and chemical stability [10]. Nitrogen-doped carbon QDs (N-CQDs) were prepared using a rapid, green, and simple microwave-assisted approach according to the procedure described in Section 3.3 (Scheme 1). When excited at 325 nm, they exhibited a distinct fluorescence emission at 417 nm. In the current study, quantitative quenching of the native fluorescence of N-CQDs caused by increasing concentrations of DCB formed the basis of the proposed spectrofluorimetric approach for the quantitative analysis of the cited drug without the need for any pre-treatment steps or high-cost instrumentation for the first time.

### 2.1. Characterization of N-CQDs

**Figure 2:**
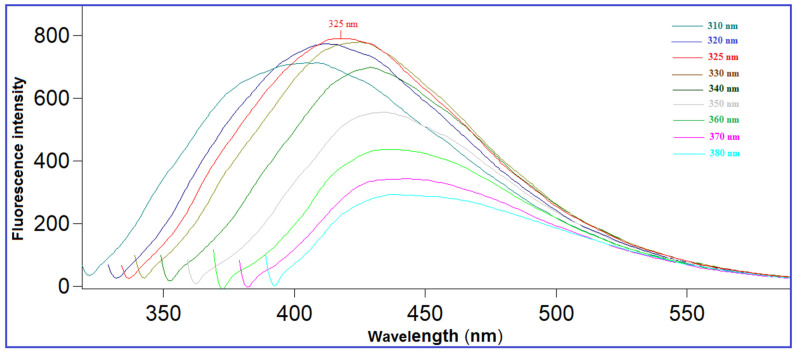
Fluorescence spectra of N-CQDs at varied excitation wavelengths (310–380 nm).

The prepared QDs were thoroughly characterized using various spectroscopic and microscopic methods. The obtained optical images of the N-CQD solution under UV and visible lights are presented in Figure S1. The solution exhibited a dark orange color under the visible light and a strong blue fluorescence when impinged with UV light. The fluorescence intensity of N-CQDs remained stable for more than four weeks. A scan of the UV spectrum was performed in order to investigate the optical characteristics of the N-CQDs, and the results are shown in Figure S2. Two characteristic bands were recorded at $\lambda_{\max}=213\;\mathrm{nm}$ and $\lambda_{\max}=275\;\mathrm{nm}$, which were attributed to the π-$\pi^{*}$ and n-$\pi^{*}$ transitions, respectively [19]. As shown in Figure S3, the N-CQDs exhibited strong fluorescence intensity at 325/417nm with a high quantum yield of 25.3%, using quinine sulfate (QS) as a reference. In addition, Figure 2 shows that the emission of synthesized QDs demonstrated excitation dependency across the 310 to 380 nm range, with the optimum fluorescence intensity recorded at the 325 nm excitation wavelength. Moreover, Figure S4 shows the images obtained by the high-resolution transmission electron microscope (HRTEM). The images show that N-CQDs were well separated without any apparent aggregation and have spherical shapes with a size in the range of 2–5 nm. The elemental analysis of the prepared N-CQDs using energy dispersive X-ray spectroscopy (EDX) was performed to ascertain the composition of the N-CQDs and confirm the level of nitrogen doping. The obtained results demonstrated that the N-CQDs were predominantly made up of C (59.21%), O (24.70%) and N (16.08%), revealing that a high nitrogen doping level was achieved (Figure S5). The Fourier transform infrared (FTIR) spectrum was also obtained to examine the surface function groups of the N-CQDs. The spectrum depicted in Figure S6 shows the following peaks: O-H/N-H ($3500-3100\;{\mathrm{cm}}^{-1}$), C-N ($2097{\;\mathrm{cm}}^{-1}$), C=O ($1701\;{\mathrm{cm}}^{-1}$), C=C ($1658\;{\mathrm{cm}}^{-1}$), and C-H ($595{\;\mathrm{cm}}^{-1}$).

### 2.2. Investigation of the Quenching Mechanism

**Figure 3:**
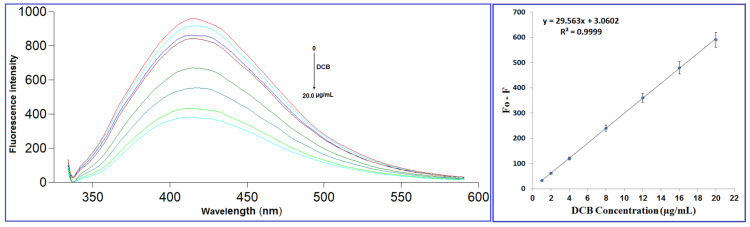
Fluorescence emission spectra of N-CQDs upon the addition of different concentrations of DCB (from top to bottom: 0, 1.0, 2.0, 4.0, 8.0, 12.0, 14.0, 16.0; 20.0 μg/mL).

As shown in Figure 3, as the DCB concentration increased, the intrinsic fluorescence of the N-CQDs quantitatively decreased, which may be attributed to the DCB’s destruction of the dot’s surface passivation layer [20]. In order to determine the potential quenching mechanism, the following Stern–Volmer Equation (1) was applied [21,22]:

(1) $$\frac{F_{0}}{F}=1+K_{sv}\left[Q\right]=1+K_{q}\tau_{0}\left[Q\right],$$

where

- F denotes the fluorescence intensity of the DCB and N-CQD mixture.
- $F_{0}$ denotes the intrinsic fluorescence intensity of the N-CQDs.
- $K_{sv}$ represents the Stern–Volmer quenching constant.
- $\left[Q\right]$ is the DCB concentration.
- $K_{q}$ denotes the quenching rate constant.
- $\tau_{0}$ represents the average lifetime of the fluorophore ($10^{-8}s$).

**Figure 4:**
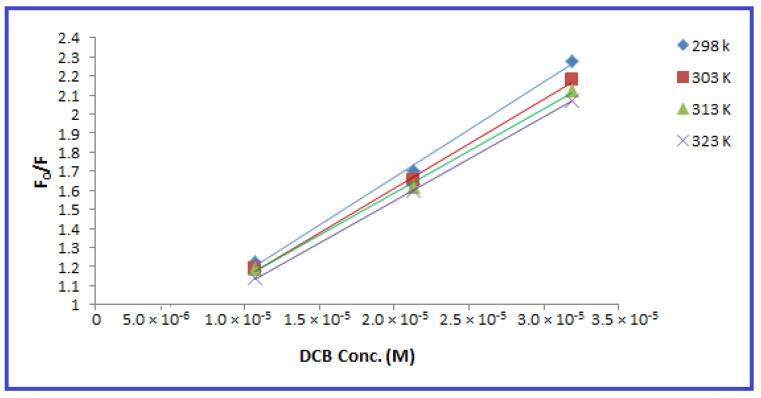
Stern–Volmer plots for the quenching of N-CQD fluorescence by DCB at four different temperature settings (298, 303, 313, and 323 K).

**Figure 5:**
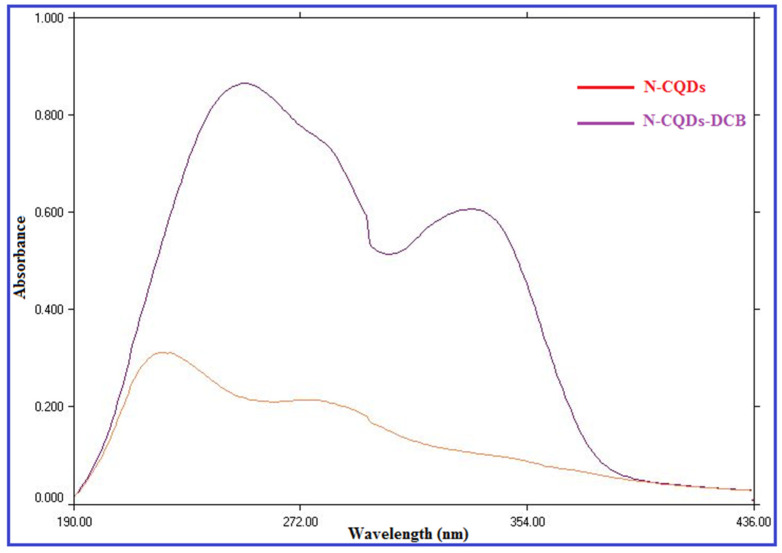
Effect of the addition of DCB on the UV absorption spectrum of N-CQDs.

**Figure 6:**
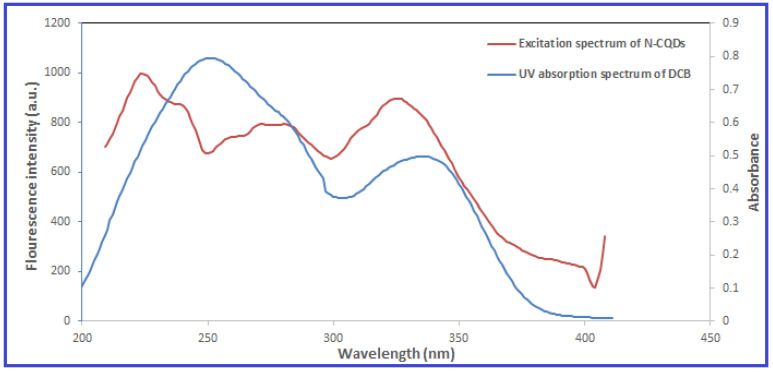
A co-plot of the excitation spectrum of the N-doped CQDs and the absorption spectrum of DCB (20.0 μg/mL).

As shown in Figure 4, the values of $K_{sv}$ at four different temperatures (298, 303, 313 and 323K) were found to be $4.97\times 10^{4}$, $4.66\times 10^{4}$, $4.40\times 10^{4}$, and $4.38\times 10^{4}L\cdot {\mathrm{mol}}^{-1}$, respectively. The decrease in $K_{sv}$ values as the temperature increases is usually an indicator of a static quenching process. The values of $K_{q}$ for the same temperatures were also calculated, and were found to be $4.97\times 10^{12}$, $4.66\times 10^{12}$, $4.40\times 10^{12}$, and $4.38\times 10^{12}L\cdot {\mathrm{mol}}^{-1}\cdot s^{-1}$. Since these values were much larger than the maximum diffusion rate constant ($2.0\times 10^{10}L\cdot {\mathrm{mol}}^{-1}\cdot s^{-1}$), the existence of the static quenching mechanism was further validated [17]. This mechanism includes the formation of N-CQD/DCB non-emissive complexes, as evidenced by the changes observed in the N-CQD UV spectra after the addition of DCB. When DCB was added, a new absorption peak appeared at 340 nm, indicating complex formation and confirming the static quenching mechanism [23] (Figure 5). Moreover, the inner filter effect was studied to investigate if it had a role in the quenching process. The overlapping between the excitation spectrum of N-CQDs and the UV–visible absorption spectrum of DCB indicated the presence of a complementary inner filter effect mechanism (Figure 6).

### 2.3. Optimization of the Experimental Parameters

According to the nature of the experiments, we decided to consider the optimization of the following two main parameters in order to achieve the maximum possible sensitivity of the suggested approach: 

**The pH of the solution:** A Britton–Robinson buffer (BRB) was used to adjust the pH level in the range of 2–12. The experiments showed that the pH had no significant effect on the determination results. Hence, no pH adjustment was required; thus, the study was conducted without the use of a buffer.

**The incubation time:** The effect of incubation time on the interaction between N-CQDs and DCB was examined and considered from 1 to 60 min. The reaction between DCB and the N-CQDs was found to be fast and occurs in less than 1 min. In addition, the fluorescence readings remained stable for more than 60 min, which adds another advantage to the proposed method.

### 2.4. Validation Studies

The validation guidelines standardized by the International Council of Harmonization (ICH) were adhered to in the validation of the proposed spectrofluorometric method [24]. Several performance criteria were evaluated, including linearity, range, limit of detection (LOD), limit of quantitation (LOQ), accuracy, precision, robustness, and selectivity.

#### 2.4.1. Linearity and Range 

The experiments considered seven ascending DCB concentrations in the range of 1.0−20.0 μg/mL to establish a relationship between the DCB concentration and the fluorescence quenching of N-CQDs (Figure 3). Linear regression was applied to the resulting measurements, leading to the following linear regression equation:

$$F_{0}-F=3.06C+29.56$$

where *F*_0_ is the fluorescence of the synthetized QDs, *F* is the fluorescence reading of the QD-DCB mixture, and *C* is the DCB concentration.

The corresponding correlation coefficient (r) was 0.9999, which indicated that the proposed method was highly linear. The analytical validation results are summarized in Table 1.

#### 2.4.2. Limit of Detection (LOD) and Limit of Quantitation (LOQ)

The sensitivity of the method was quantified by means of two measures, the limit of detection (LOD) and limit of quantification (LOQ), which were calculated as follows:

$$\mathrm{LOD}=3.3S_{a}/b,$$

and

$$\mathrm{LOQ}=10S_{a}/b,$$

where $S_{a}$ and b denote the standard deviation of the intercept and the slope of the regression line, respectively. 

As presented in Table 1, the LOD and LOQ were 0.11 and 0.33 µg/mL, respectively, indicating the acceptable sensitivity of the proposed procedure.

#### 2.4.3. Accuracy and Precision

Accuracy refers to how close a measurement is to the true or accepted value, while precision refers to how close measurements are to each other. The mean percentage recovery was measured over the considered DCB concentration range. As listed in Table 2, the recorded recovery percentages were relatively high (98.50−100.83%), indicating that the proposed method is sufficiently accurate. 

As for the method’s precision, multiple measurements were taken within the same day and across three consecutive days. For each of the intra- and inter-day experiments, three distinct DCB concentration levels were considered. The used concentrations were 2.0, 8.0 and 16.0 μg/mL. The obtained relative standard deviation (RSD) and error percentages are listed in Table 3. The obtained values were sufficiently small (% RSD (less than 1.185) and % error (less than 0.68)), indicating the satisfactory precision of the proposed method.

#### 2.4.4. Robustness

The robustness of the method was studied, where the effect of minor variations in the volume of N-CQDs (125.0 μL ± 1) on the fluorescence sensing of DCB was investigated. It was verified that small changes did not significantly affect the quenching of the N-CQD fluorescence intensities by DCB, indicating the robustness of the developed method (Table 4).

#### 2.4.5. Selectivity

**Figure 7:**
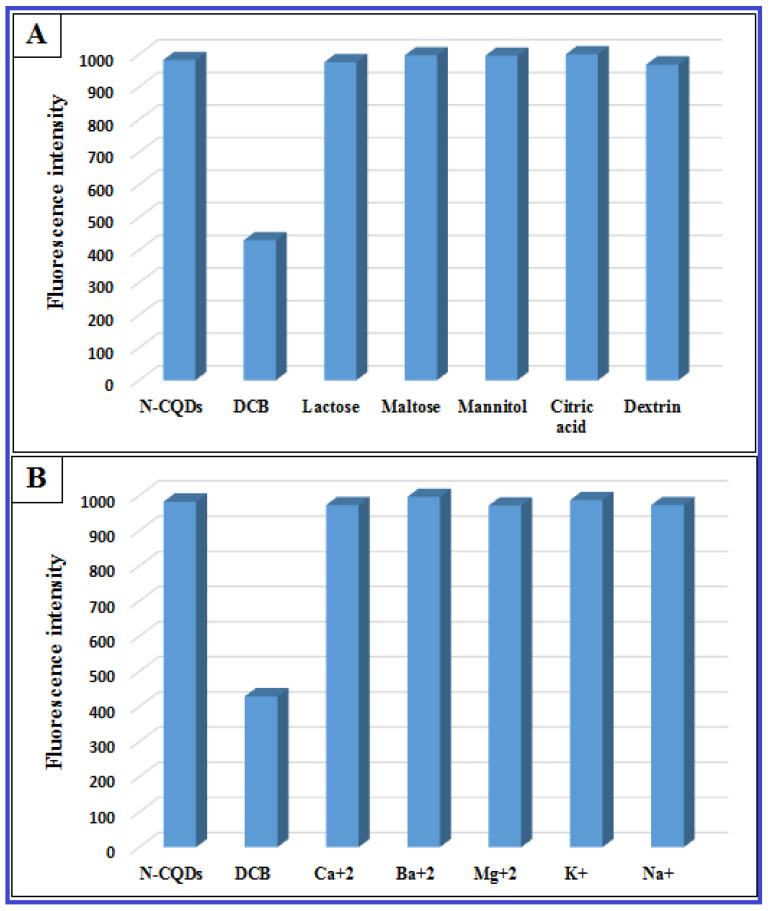
(**A**) The response of possible interfering excipients; (**B**) the selectivity of N-CQDs towards DCB in the presence of different metal ions.

Finally, the selectivity of the proposed approach was evaluated by investigating its ability to determine DCB in the commercial Tablets (Vizimpro^®^ tablets) with low %RSD (less than 1.131%) and high % recovery (98.29–101.46%), without any interference from the existing excipients (Table 5). The possible interfering excipients, such as lactose, maltose, mannitol, dextrin, and citric acid, were studied in detail and confirmed the high selectivity of the method, since they minimally affected the fluorescence intensity of the N-CQDs (Figure 7A). Similarly, the method selectivity was proven by its ability to detect the studied drugs in the presence of different metal ions such as Na^+^, K^+^, Ca^+2^, Mg^+2^, and Ba^+2^ without any interference (Figure 7B). In addition, the proposed method was able to determine DCB in the presence of other anticancer drugs, including larotrectinib and palbociclib. The tolerance limit of these drugs was determined as the concentration that results in 2% or higher relative errors [6], which was found to be 1.0 µg/mL for both drugs. Accordingly, the developed method showed excellent selectivity for the determination of DCB without any interference.

### 2.5. Application in Pharmaceutical Preparations

The proposed method was efficiently used to determine DCB in its commercial tablet formulation (Vizimpro^®^ tablets) with high selectivity and without any interference from the existing excipients, including lactose, maltose, mannitol, dextrin, and citric acid, in addition to the excipients present in the film coating. By analyzing different concentrations of the tablet extract, low %RSD (less than 1.131%) and high % recovery (98.29–101.46%) values were obtained, as detailed in Table 5. 

## 3. Experimental Procedure

### 3.1. Instrumentation and Tools

The following is a list of the instruments and tools used in the current study:

- Fluorescence measurements were obtained using the Cary Eclipse Fluorescence Spectrophotometer from Agilent Technologies (Santa Clara, CA, USA), which operated with a Xenon flash lamp at 750 V.
- The Jenway pH meter 3510 (Jenway, London, UK) was used to perform all the pH measurements.
- The Nicolet iS10 Fourier transform infrared (FTIR) spectrometer from ThermoFisher Scientific (Waltham, MA, USA) was used to obtain the required FTIR spectra. Measurements were taken for 32 scans with a resolution of 4 cm^−1^. The device has a 4000 to $1000\;{\mathrm{cm}}^{-1}$ DTGS detector, along with a Ge/KBr beam splitter.
- The light absorbance of the analytes was measured by a double-beam spectrophotometer (PG Instrument, Wibtoft, UK).
- Transmission electron microscopy (TEM) and energy dispersive X-ray spectroscopy (EDX) were performed using the JEM-2100 high-resolution transmission electron microscope (HRTEM) by JEOL (Tokyo, Japan), which operated at 200 kV.
- Sigma 2-16P (Germany) benchtop cooling centrifuge.
- VM-300P vortex mixer from Gemmy Industrial Corp (Taiwan).
- Membrane filters with a pore size of 0.45 μm purchased from Phenomenex (Torrance, CA, USA).
- S-101H ultrasonic bath from Sonicor Inc. (West Babylon, NY, USA).
- Domestic Microwave (GE614ST, 2800 W, 2450 MHz, Samsung, Kuala Lumpur, Malaysia).

### 3.2. Materials and Stock Solutions

All the materials and reagents used in the experimental part of this study were of analytical grade. In addition, double distilled water was used throughout the study. The following is a list of the most important materials and solutions used in the experiments:

- Dacomitinib (%purity 99.89) was obtained from the European division of Pfizer (Europe MA EEIG, Brussels, Belgium).
- Vizimpro^®^ film-coated tablets (labeled to contain DCB at a concentration of 30 mg/tablet) were also obtained from Pfizer (Europe MA EEIG, Brussels, Belgium).
- Urea was purchased from Sigma-Aldrich (St. Louis, MO, USA).
- Methanol was acquired from Tedia (Fairfield, OH, USA).
- Navel orange (species *Citrus sinensis*) was obtained from a local Egyptian market.
- A 100.0 μg/mL stock solution of DCB was prepared in methanol. Subsequent dilutions were formed using double distilled water and the solution was found to be stable for at least 14 days at 4 °C.
- A Britton–Robinson buffer (BRB) with a concentration of 0.2 M was prepared in distilled water with different pH levels in the range of 2–12.

### 3.3. Procedure for the Synthesis of the Doped N-CQDs

The synthesis procedure of N-CQDs started by dissolving 3 gm of urea into 50 mL of orange juice (navel orange, species *Citrus sinensis*). The solution was heated in a domestic microwave for 10 min until it was completely charred. After being cooled, the product was diluted with distilled water to 100 mL and placed into the centrifuge for 15 min at 6000 rpm. This removed any suspended particles present in the charred mixture. The clear supernatant lying on top of the residue was filtered and distilled water was added to reach a volume of 200 mL to produce the N-CQD stock solution. The working solution was then obtained by placing 10 mL of the prepared stock solution into a volumetric flask and double distilled water was added to reach a volume of 100 mL. The solutions were stored in the refrigerator for further use (Scheme 1).

### 3.4. Spectrofluorimetric Measurements

After the optimization of different parameters, 125 μL portions of the N-CQD solution were deposited into a set of 5 mL volumetric flasks, along with the appropriate varying aliquots of the DCB drug to yield a concentration in the range of 1.0−20.0 µg/mL. The flasks were then completed with distilled water. After excitation at 325 nm, the spectrofluorimetric measurements were taken at the emission wavelength of 417 nm. The calibration curve was constructed by plotting the fluorescence quenching against the drug concentration and linear regression analysis was carried out to analyze the curve. 

### 3.5. Quantum Yield Measurements

The photoemissive efficiency was measured using the quantum yield (QY) percentage, denoted by Φ. For the N-CQDs used in this study, the quantum yield was determined using the following equation [25]:

$$\Phi_{N-\mathrm{CQDs}}=\Phi_{\mathrm{QS}}\times \left(F_{N-\mathrm{CQDs}}/F_{\mathrm{QS}}\right)\times {\left(\eta_{N-\mathrm{CQDs}}/\eta_{\mathrm{QS}}\right)}^{2}\times \left(A_{\mathrm{QS}}/A_{N-\mathrm{CQDs}}\right),$$

where the quantities F, η, and A denote the integrated emission intensity, the solvent refractive index, and the absorbance, respectively. The subscript QS is an abbreviation for quinine sulfate, which was used as the standard in the calculations. The QS was dissolved in 0.1 M of H_2_SO_4_ and had the quantum yield $\Phi_{\mathrm{QS}}=0.54$ at 350 nm. In the aqueous solutions, the refractive indices were equal, leading to $\eta_{N-\mathrm{CQDs}}/\eta_{\mathrm{QS}}=1$. The absorbance was kept below 0.1 in order to reduce the absorption effect. In the current method, A_QS_ and A_N-CQDs_ were found to be 0.02 and 0.059.

### 3.6. Application of the Proposed Method to Vizimpro^®^ Tablets

The proposed spectrofluorometric determination method was applied to the commercial Vizimpro^®^ tablets. In the experiments, 10 tablets were grinded and thoroughly mixed. Then, an amount of the powder equivalent to 10 mg of DCB was deposited into a 100 mL volumetric flask. A 50 mL portion of methanol was added to the flask and the mixture was sonicated in the S-101H ultrasonic bath for 15 min before completing the volume of the flask to the mark. The resulting methanolic solution was filtered and appropriate portions were placed into 5 mL volumetric flasks, which were then filled with double distilled water. The flask contents were subjected to the spectrofluorimetric procedure described in Section 3.4. Percentage recoveries were calculated based on the regression equation, or directly taken from the calibration curve.

## 4. Conclusions 

Dacomitinib has recently been approved by the United States FDA as a first-line treatment for patients with metastatic NSCLC. Due to the unavailability of a simple, cost-effective, and environmentally friendly validated determination method for DCB, the current study sought to develop the first spectrofluorimetric approach for its determination, using an N-CQD-based fluorescent probe. The quenching of the native fluorescence of quantum dots is a promising technique for the determination of different chemical species. The driving force behind the choice of N-CQDs was the multitude of advantages reported in the literature, including low toxicity, low cost, and simple synthesis procedures. N-CQDs were synthesized through a simple, green, and rapid microwave-assisted technique in less than 10 min. The proposed method was based on the quantitative and selective fluorescence quenching effect of DCB on the N-CQDs’ native fluorescence. The results showed a linearly proportional relationship between the fluorescence quenching and the DCB concentration. The proposed approach demonstrated sufficient sensitivity with an LOD of 0.11 µg/mL. In addition, the quenching mechanism was investigated and the results suggested that the quenching was of a static nature with a complementary inner filter effect. The proposed method was also deemed to be eco-friendly because it reduced the use of organic solvents and the synthetic approach was based on using orange juice as a natural starting material. The proposed method was validated in accordance with ICHQ2 (R1) guidelines. It was also applied in the analysis of the commercial tablet dosage form of the DCB with high accuracy and repeatability. Hence, the developed method represents a good candidate for real applications in quality control laboratories. 

## Figures and Tables

**Table 1:** Validation data for the determination of DCB by the proposed spectrofluorimetric method.

Parameter	DCB
λ_ex_–λ_em_	325–417 nm
Concentration range (μg/mL)	1.0–20.0
Slope	29.56
Intercept	3.06
Correlation coefficient (r)	0.9999
S.D. of residuals (S_y/x_)	2.12
S.D. of intercept (S_a_)	0.99
S.D. of slope (S_b_)	0.11
Percentage relative standard deviation, % RSD	0.98
Percentage relative error, % Error	0.37
Limit of detection, LOD ^a^ (μg/mL)	0.11
Limit of quantitation, LOQ ^b^ (μg/mL)	0.33

^a^ LOD = 3.3 *S_a_*/*b*; ^b^ LOQ = 10 *S_a_*/*b*, where *S_a_* = standard deviation of the intercept and *b* = slope.

**Table 2:** Accuracy data of the designed method for the determination of DCB in pure form.

Parameter	DCB		
	**Amount Taken (μg/mL)**	**Amount Found (μg/mL)**	% Found *
	1.0	0.99	98.50
	2.0	1.98	99.00
	4.0	3.96	98.98
	8.0	8.01	100.19
	12.0	12.07	100.62
	16.0	16.13	100.83
	20.0	19.85	99.27
Mean			99.69
± S.D.			0.98
% RSD			0.984
% Error			0.371

* Average of 3 separate determinations.

**Table 3:** Intra- and inter-day precision data for the determination of DCB by the proposed method.

DCB		**Intra-Day ^a^**			**Inter-Day ^b^**		
	**Conc. (μg/mL)**	$\bar{x}$ **± S.D**	**% RSD**	**% Error**	$\bar{x}\;$ **± S.D**	**% RSD**	**% Error**
	2.0	99.02 ± 0.73	0.737	0.42	98.71 ± 1.17	1.185	0.68
	8.0	99.85 ± 0.96	0.961	0.55	100.23 ± 0.79	0.788	0.46
	16.0	100.13 ± 0.88	0.878	0.51	99.84 ± 0.96	0.961	0.55

Each reading is the average of three separate determinations; ^a^ within the day; ^b^ three consecutive days.

**Table 4:** Evaluation of the robustness of the proposed method.

Factor Variation	DCB	
1. Volume of N-CQDs (125.0 μL ± 1)	% Recovery *	% RSD
124.0 µL	98.87	0.94
125.0 µL	99.42	0.86
126.0 µL	100.21	1.16

* Each result is the average of 3 separate determinations.

**Table 5:** Determination of DCB in Vizimpro^®^ tablets by the proposed spectrofluorimetric method.

Parameter	DCB		
Vizimpro^®^ Tablets (30 mg DCB/Tablet)	**Amount Taken** **(μg/mL)**	**Amount Found** **(μg/mL)**	% Found *
	4.0	3.93	98.29
	8.0	7.99	99.95
	12.0	12.17	101.46
	16.0	15.94	99.60
	20.0	19.96	99.81
Mean			99.82
± S.D.			1.13
% RSD			1.131
% Error			0.505

* Each result is the average of 3 separate determinations.

## Data Availability

Data available upon request to the corresponding author.

## Supplementary Materials

The following supporting information can be downloaded at: https://www.mdpi.com/article/10.3390/molecules28052351/s1, Figure S1: Optical images of the prepared N-CQDs under (a) visible light, and (b) UV light; Figure S2: UV absorption spectrum of N-CQDs; Figure S3: Fluorescence spectra of N-CQDs; (a) excitation and (b) emission spectra; Figure S4: The typical HRTEM image of N-CQDs; Figure S5: TEM-EDX analysis of the prepared N-CQDs; Figure S6: FTIR spectrum presenting the surface functionality of N-CQDs.

## Sample Availability

Samples of the compounds are available from the authors.
