# Supplementary material for: A Novel Quantum Dots-Based Fluorescent Sensor for Determination of the Anticancer Dacomitinib: Application to Dosage Forms
Source: Molecules. 2023 Mar 3;28(5):2351. doi: 10.3390/molecules28052351 (PMC10005270; doi:10.3390/molecules28052351)
Supplement: Supplementary file 1 [file molecules-28-02351-s001.zip › molecules-2231870-supplementary.pdf]

Supplementary Materials

A Novel Quantum Dots-Based Fluorescent Sensor for Determination of the Anticancer Dacomitinib: Application to Dosage Forms

Manal A. Alossaimi [1,*], Heba Elmansi [2], Mai Alajaji [3], Ali Altharawi [1], Abdulmalik S. A. Altamimi [1] and Galal Magdy [4]

[1] Pharmaceutical Chemistry Department, College of Pharmacy, Prince Sattam Bin Abdulaziz University, Al-Kharj 11942, Saudi Arabia
[2] Pharmaceutical Analytical Chemistry Department, Faculty of Pharmacy, Mansoura University, Mansoura P.O. Box 35516, Egypt
[3] King Abdullah International Medical Research Center, College of Pharmacy, King Saud bin Abdulaziz University for Health Sciences, Riyadh 14611, Saudi Arabia
[4] Pharmaceutical Analytical Chemistry Department, Faculty of Pharmacy, Kafrelsheikh University, Kafrelsheikh P.O. Box 33511, Egypt
* Correspondence: m.alossaimi@psau.edu.sa

Supplementary Figures

Figure S1. Optical images of the prepared N-CQDs under (a) visible light, and (b) UV light

Figure S2. UV absorption spectrum of N-CQDs

Figure S3. Fluorescence spectra of N-CQDs; (a) excitation and (b) emission spectra

Figure S4. The typical HRTEM image of N-CQDs

Figure S5. TEM-EDX analysis of the prepared N-CQDs

Figure S6. FTIR spectrum presenting the surface functionality of N-CQDs

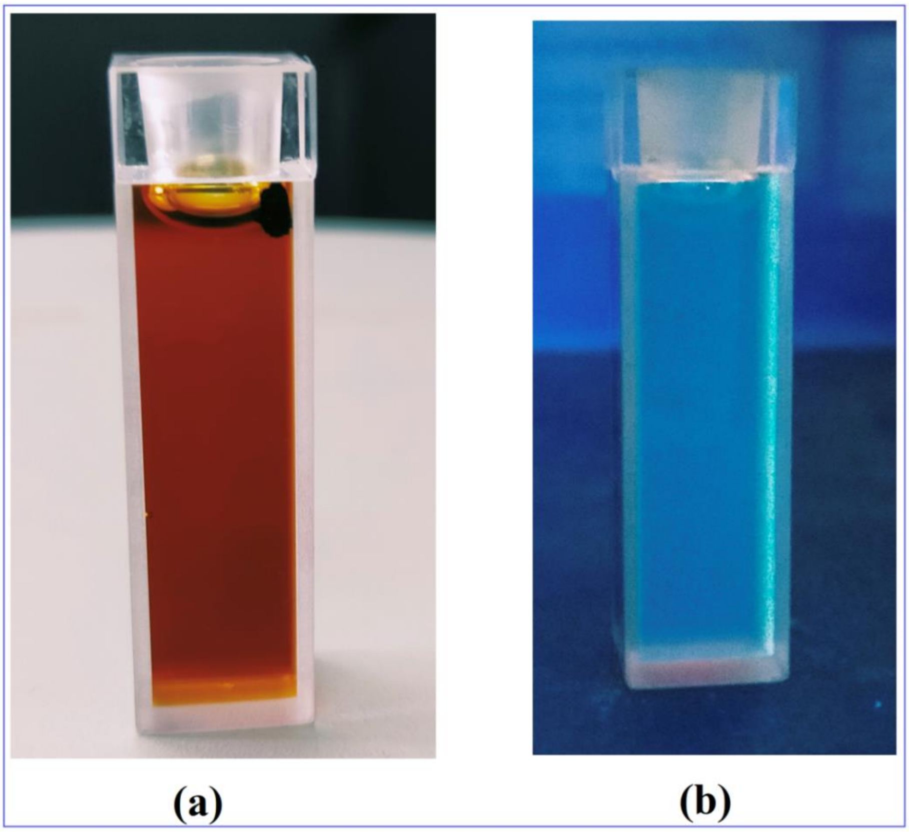

Figure S1. Optical images of the prepared N-CQDs under (a) visible light, and (b) UV light.

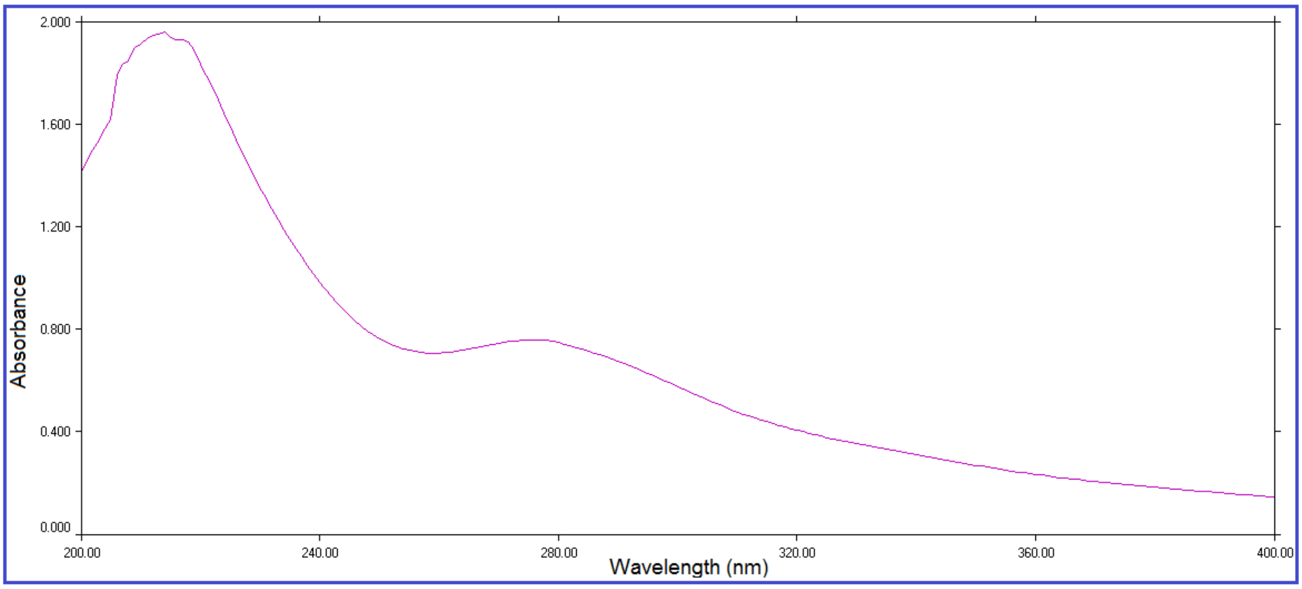

Figure S2. UV absorption spectrum of N-CQDs.

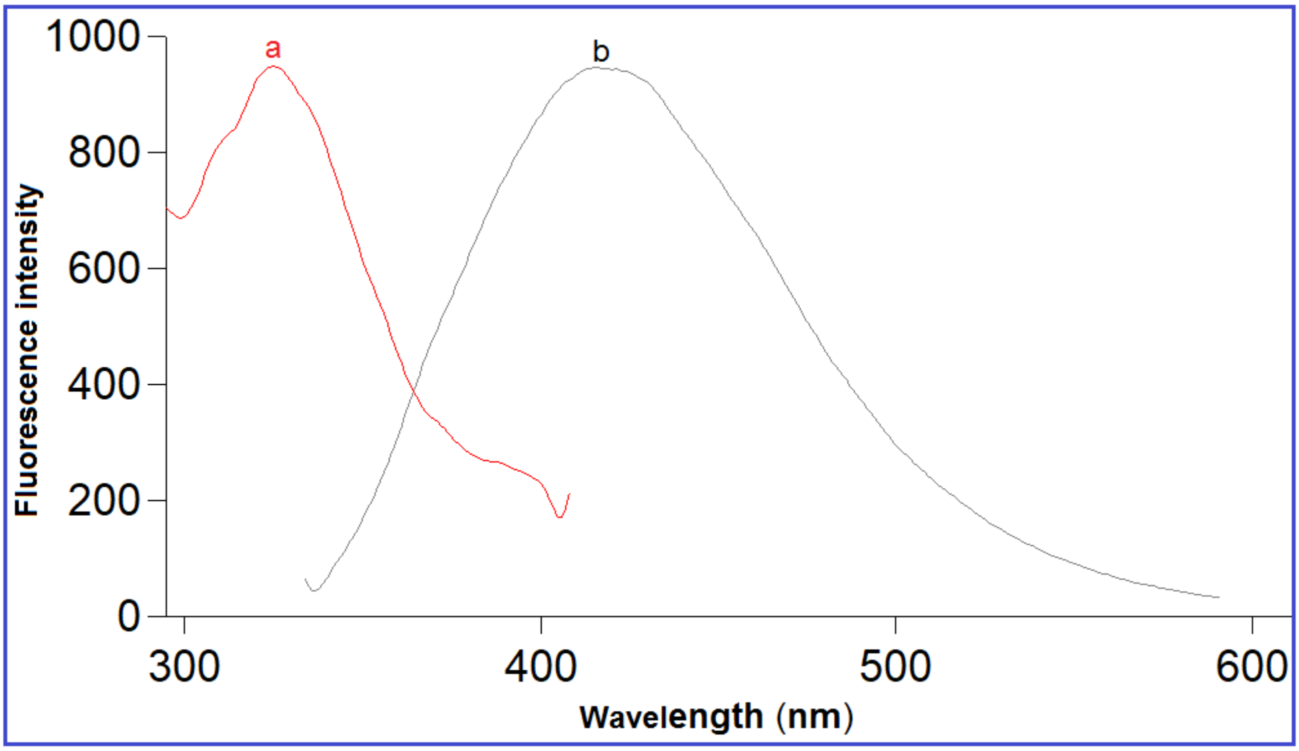

Figure S3. Fluorescence spectra of N-CQDs; (a) excitation and (b) emission spectra.

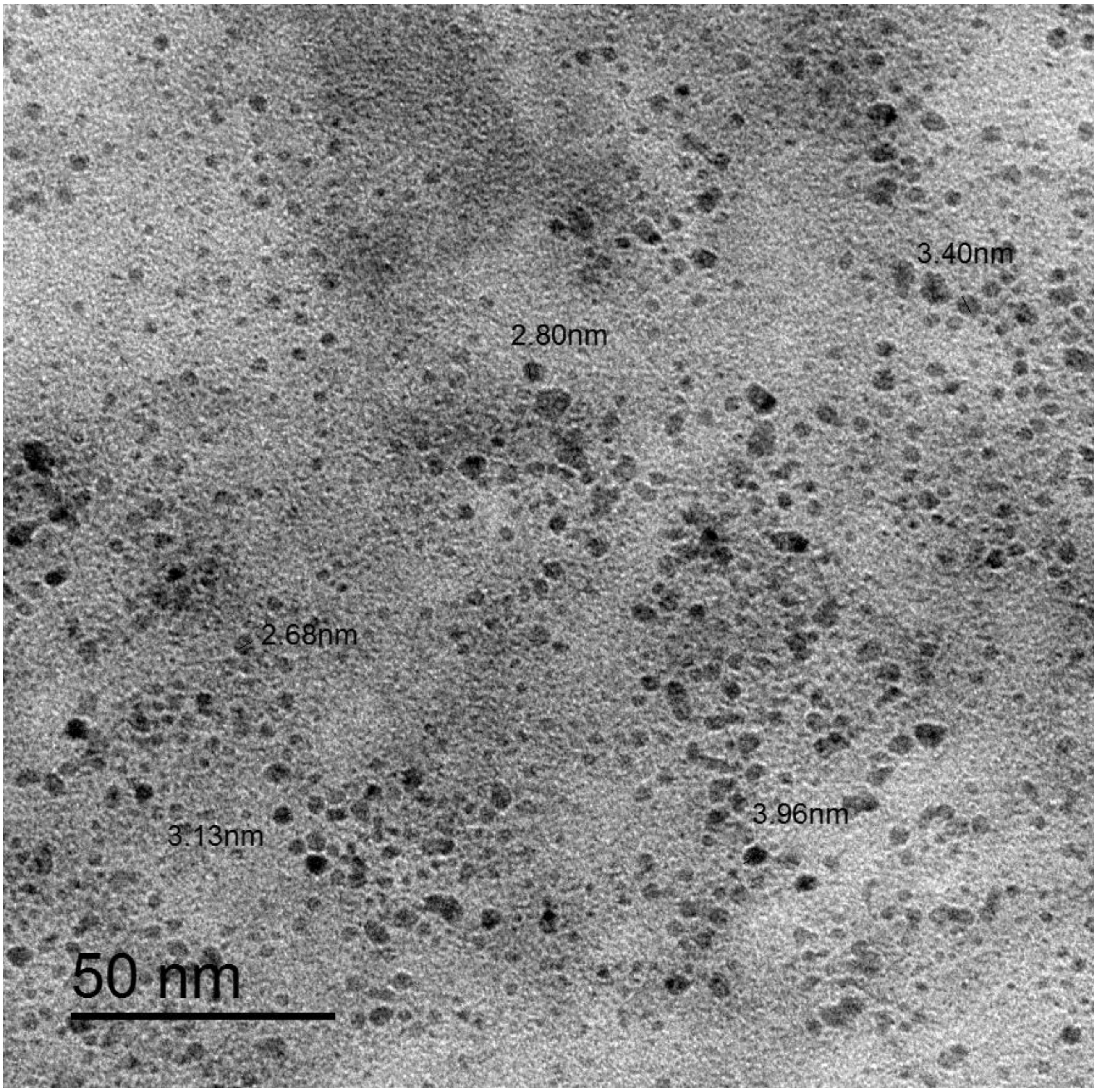

Figure S4. The typical HRTEM image of N-CQDs.

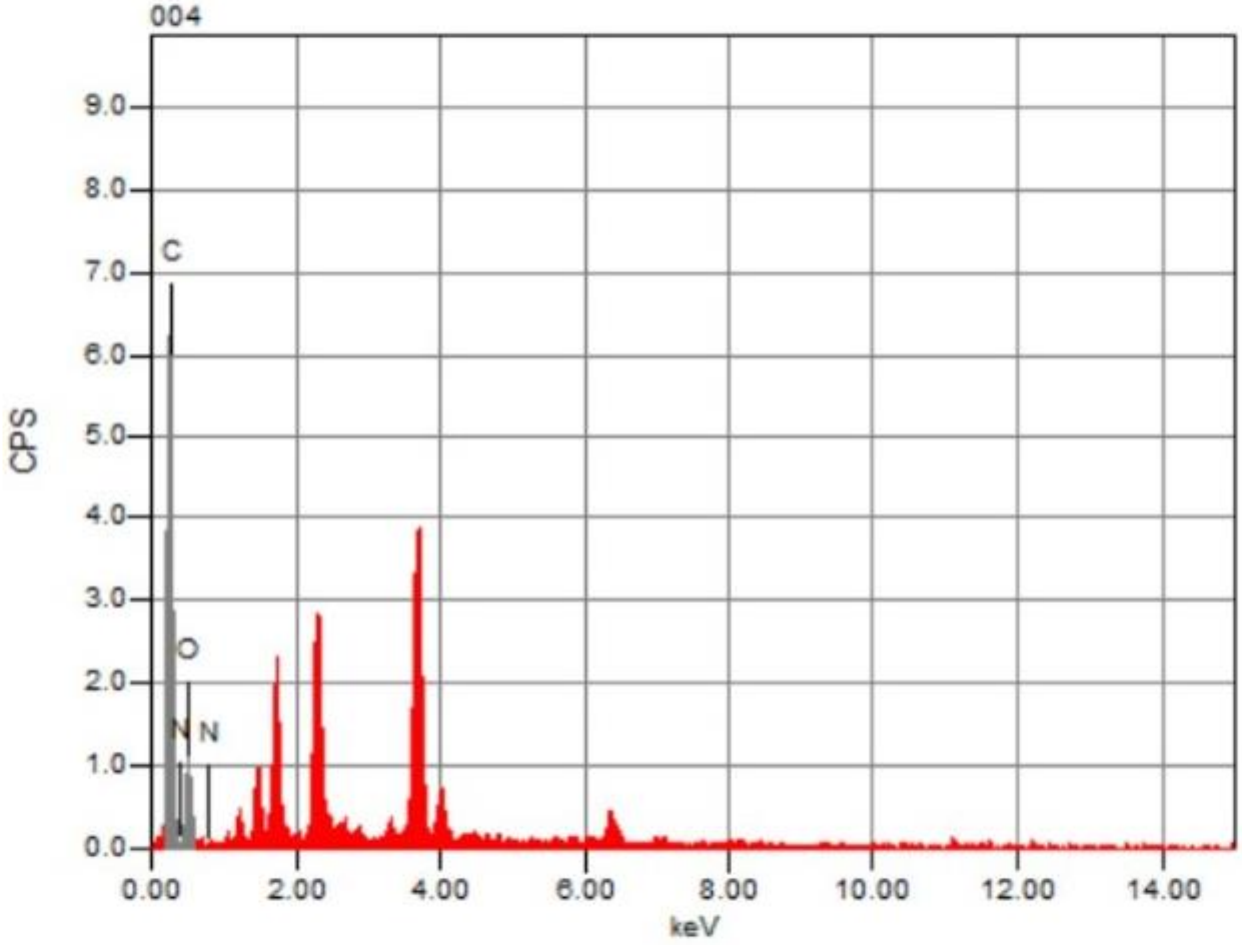

Figure S5. TEM-EDX analysis of the prepared N-CQDs.

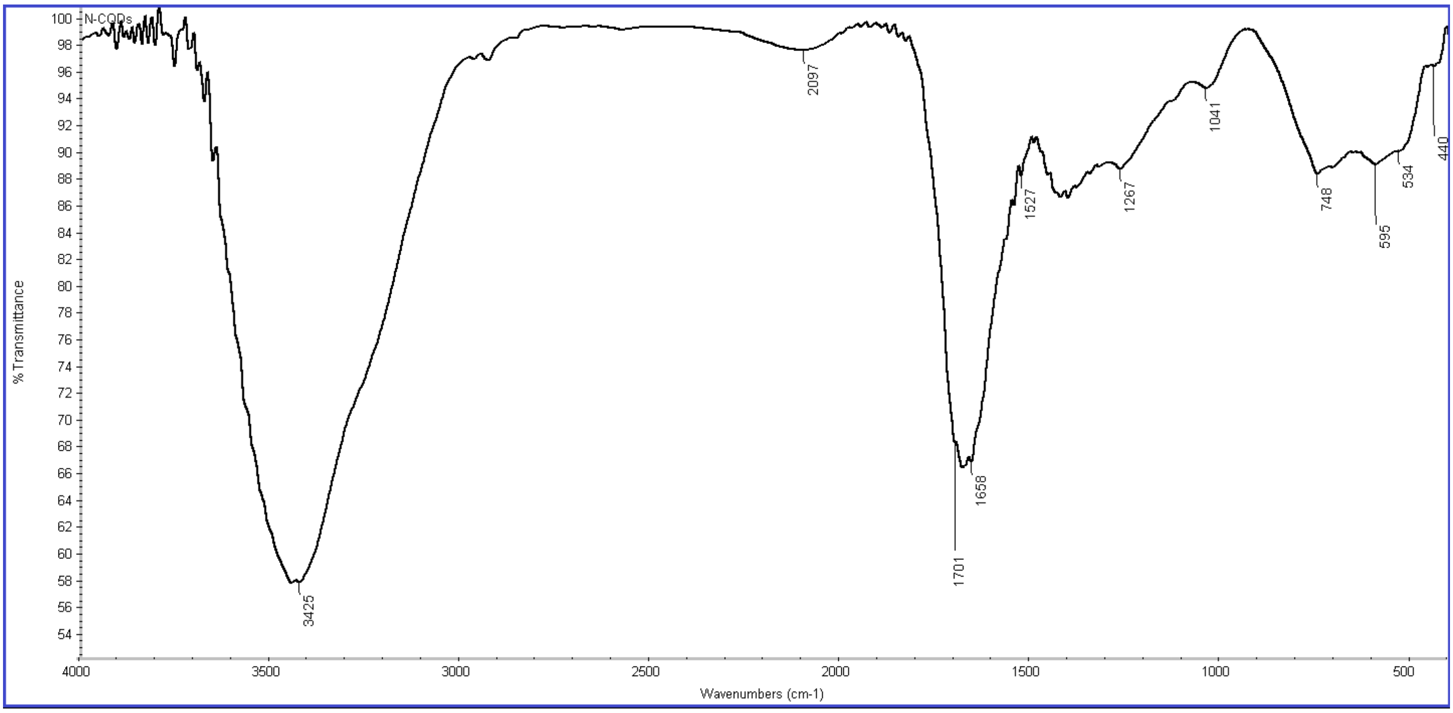

Figure S6. FTIR spectrum presenting the surface functionality of N-CQDs.
